# Prognostic significance of promoter DNA hypermethylation of the cysteine dioxygenase 1 (CDO1) gene in primary gallbladder cancer and gallbladder disease

**DOI:** 10.1371/journal.pone.0188178

**Published:** 2017-11-21

**Authors:** Kazuharu Igarashi, Keishi Yamashita, Hiroshi Katoh, Keita Kojima, Yosuke Ooizumi, Nobuyuki Nishizawa, Ryo Nishiyama, Hiroshi Kawamata, Hiroshi Tajima, Takashi Kaizu, Yusuke Kumamoto, Masahiko Watanabe

**Affiliations:** Department of Surgery, Kitasato University Hospital, Minami-ku, Sagamihara, Kanagawa, Japan; University of South Alabama Mitchell Cancer Institute, UNITED STATES

## Abstract

**Background:**

Aberrant promoter DNA methylation of the cysteine dioxygenase 1 (CDO1) gene is found in various human cancers and is associated with clinical outcome. In this study, we assessed for the first time the clinicopathological significance of *CDO1* methylation in primary gallbladder cancer (GBC) in comparison with non-malignant gallbladder disease.

**Methods:**

*CDO1* DNA methylation was quantified using quantitative TaqMan methylation specific PCR (Q-MSP) in 99 primary GBC patients together with the 78 corresponding non-tumor tissues and 26 benign gallbladder disease (including 7 patients with xanthogranulomatous cholecystitis) who underwent surgical resection between 1986 and 2014.

**Results:**

The average *CDO1* TaqMeth value of primary GBCs was 23.5±26. These values were significantly higher than those of corresponding non-tumor tissues (average 8±13, p < .0001) and diseased gallbladder tissues from patients with benign gallbladder diseases (average 0.98±1.6, p < .0001). *CDO1* hypermethylation is also found in xanthogranulomatous cholecystitis. Using a cut-off value of 17.7, GBC cases with *CDO1* hypermethylation (n = 47) showed significantly poorer prognosis than those with *CDO1* hypomethylation (n = 52) (p = 0.0023). Multivariate Cox proportional hazards analysis identified that *CDO1* hypermethylation was an independent prognostic factor. Notably, *CDO1* hypermethylation showed prognostic relevance, especially in stage II GBC, in which it is highly anticipated to work as a predictive marker for candidates of adjuvant therapy.

**Conclusions:**

Promoter NA methylation of *CDO1* was demonstrated for the first time to be a cancer-associated methylation in primary GBC, and it has the potential to be a prognostic biomarker of GBC for high-risk patients with stage II GBC.

## Introduction

Cancer statistics predicted an estimated 11,420 new gallbladder cancer (GBC) cases and 3,710 GBC deaths in the United States in 2016 [1]. GBC has been associated with a poor prognosis, and the depth of tumor invasion and the presence of lymph node metastasis have been reported to be important prognostic factors [2]. High rates of both local and distant recurrence have prompted interest in the use of adjuvant chemotherapy and radiation therapy (RT). At present, however, there is a paucity of high-quality evidence to support adjuvant treatment in GBC, and patients should be encouraged to participate in clinical trials evaluating new strategies. Therefore, molecular biomarkers are highly demanded in the clinic for tumor diagnosis and prognosis prediction, with establishment of an appropriate operative extent and optimal guidelines of postoperative adjuvant therapy.

Epigenetic gene silencing of tumor suppresser genes through promoter DNA hypermethylation is a common feature in human cancers, whereas cancer specific methylation is a relatively rare event [3, 4]. We have developed pharmacologic reversal of epigenetic silencing and thereby uncovered a myriad of transcriptionally repressed genes in human cancers [5]. Using this technique, we have identified novel tumor suppressor gene candidates including the cysteine dioxygenase type 1 (CDO1) gene in human cancers. Aberrant *CDO1* promoter DNA methylation in breast cancer has firstly been reported by Dietrich et al. as a prognostic biomarker in patients who received adjuvant chemotherapy [6]. The association of *CDO1* methylation with overall survival in breast cancer patients was further confirmed by Jeschke et al [7]. Furthermore, promoter DNA of the CDO1 gene was frequently methylated in esophagus, lung, bladder, gastric, and colorectal cancers [8]. Additionally, we and others investigated strong association of CDO1 gene promoter DNA methylation with poor prognosis in primary breast cancer [9], renal clear-cell cancer [10], esophageal squamous cell carcinoma [11] and Barrett esophagus adenocarcinoma [12]. Intriguingly, some previous literature has shown the availability of this methylation for diagnosis using a liquid biopsy for analysis of *CDO1* promoter DNA methylation [13, 14]

In this study, we investigated for the first time the clinicopathological and prognostic relevance of promoter DNA methylation of the CDO1 gene in primary GBC in comparison with gallbladder disease.

## Material and methods

### GBC cell lines and tissue samples

We used 4 different human cancer cell lines in this study. The GBC cell lines (G-415 and TGBC2TKB), colorectal cancer (CRC) cell line DLD1 and the hepatoblastoma cell line HepG2 were purchased from RIKEN Bio Resource Centre (Ibaraki, Japan). DLD1 and HepG2 were used as positive and negative controls of methylation, respectively. DLD1 and G-415 were maintained in RPMI 1640 Medium (GIBCO, Carlsbad, CA) and HepG2 and TGBC2TKB were maintained in DMEM (Sigma Aldrich, St. Louis, Mo), containing 10% fetal bovine serum and Penicillin-Streptomycin (GIBCO).

We retrospectively recruited 99 primary gallbladder cancer (GBC) patients and 26 benign gallbladder disease patients, including 10 chronic cholecystitis patients (CGC), 9 adenomyomatosis patients (ADM), 7 xanthogranulomatous cholecystitis patients (XGC), who had undergone surgical resection of the primary tumors at the Kitasato University Hospital between June 1986 and September 2014. We included cases with distant metastasis or residual diseases. All 99 GBC patients had complete information available for all of the clinicopathological factors.

We extracted DNA from the formalin-fixed paraffin embedded (FFPE) 99 tumor tissues (T) and 78 corresponding non-cancerous mucosa tissues (CN) of the 99 primary GBC patients. In addition, 26 diseased gallbladder tissues (NN) from patients with benign gallbladder disease were included to compare methylation status. All tissue samples were collected at the Kitasato University Hospital, all patients had agreed to the use of their pathological specimens and written consent was obtained from all patients and healthy donors before sample collection. The present study was approved by the Ethics Committee of Kitasato University.

### Clinicopathological factors

TNM classification was performed according to the 6^th^ edition of the Japanese Society of Hepato-Biliary-Pancreatic Surgery (JSHBPS).

Operative procedures were as follows: Surgical resection was divided into 3 groups depending on the degree of resection; simple cholecystectomy (SCx), the so-called standard resection (SR), and extended resection (ER). SR refers to cholecystectomy plus liver bed resection approximately 2 cm from the gallbladder or partial resection of the liver, the lower part of segment 4 and 5. ER included the following operative procedures: hepatectomy more than segmentectomy; pancreaticoduodenectomy; and hepatopancreaticoduodenectomy.

Postoperative chemotherapy was as follows: Adjuvant therapy after surgical resection was not strictly protocol-driven and was administered in the treatment of patients at the discretion of surgeons and oncologists. The chemotherapy regimens consisted of 5-fluorouracil (5-FU)-based chemotherapy (5-FU only, or 5-FU/mitomycin-C by venous infusion, and tegafur/uracil (UFT) or 5’-deoxy-5-fluorouridine (5’DFUR), or TS-1 as oral therapy). Others were mitomycin-C or gemcitabine or gemcitabine + TS-1.

### RNA extraction and reverse transcription-polymerase chain reaction (RT-PCR)

Total RNA from cell lines were extracted using Rneasy Mini Kit (QIAGEN, Hilden, Germany). Reverse-transcribed with Super Script III reverse transcriptase kit (Invitrogen, Carlsbad, CA). Primers sequences are also included in S1 Table. RT-PCR was performed by 30 cycles of 95°C for 1 min, 58°C for 1 min, and 72°C for 1 min., and the PCR products were separated on 1.5–2.0% agarose gel, then visualized by ethidium bromide staining. β-actin was used as an internal control.

### 5-Aza-dC and TSA treatment

Cells (1×10^6^ cells/T-75 flask) were treated with 1 or 5 μM of the demethylating agent 5-aza-2′-deoxycytidine (5-Aza-dC) (Sigma-Aldrich) dissolved in 50% acetic acid or mock-treatment with PBS including the same amount of acetic acid every 24 hr for 4 days. When combined with the histone deacetylase inhibitor trichostatin A (TSA) (Sigma-Aldrich), 300 nM TSA was added to the medium for the final 24 hr.

### Bisulfite treatment of DNA

Genomic DNA of FFPE and cell lines were extracted using QIAamp DNA FFPE Tissue Kit and QIAamp DNA Mini Kit (QIAGEN). Bisulfite treatment was done by using a Methylation-Gold Kit (QIAGEN).

### Quantitative-methylation-specific PCR (Q-MSP)

Quantitative-TaqMan methylation specific PCR (Q-MSP) was carried out using iQ Supermix (Bio-Rad Laboratories, Hercules, CA) in triplicate on the C1000 Touch^™^ Thermal Cycler CFX96 Real Time System (Bio-Rad). PCR conditions and the primer sequences are provided in S1 Table. Serial dilutions of bisulfite modified DNA from the CRC cell line DLD1 was used to construct the calibration curve on each plate as a methylation positive control, and the Hepatoblastoma cell line HepG2 was used as a negative control. The methylation value (designated as the TaqMeth value as previously described) was defined by the ratio of the amplified signal value of methylated *CDO1* to the value for β-actin, which was then multiplied by 100. This ratio was used as a measure for the relative level of methylated DNA in samples. Many literatures previously assessed CpG islands structure and bisulfite-sequencing in the *CDO1* gene promotor and analysis of *CDO1* promotor activity by luciferase reporter assay to investigate whether *CDO1* expression is regulated by the promotor methylation [7–9].

### Immunohistochemistry

Formalin fixed, paraffin-embedded histological sections (3 μm thick) were immunostained using the anti-CDO1 rabbit polyclonal antibody (dilution of 1:100) (ATLAS ANTIBODIES, Bromma, Sweden) at 4°C overnight. The primary antibody was visualized using the Histofine Simple Stain PO (M) kit (Nichirei, Tokyo, Japan). And immune complexes were detected using the 3,3′-diamino-benzidine tetrahydrochloride (DAB) substrate, as a chromogen for 2 minutes. Sections were counterstained in Mayer’s Hematoxyline.

### Statistical analysis

Student’s *t*-test was used for analysis of continuous variables, and the χ^2^ test was used for analysis of categorical variables. Clinicopathological characteristics and follow up data were analyzed in terms of overall survival (OS). The follow up time was calculated from the date of surgery to death. OS was calculated by the Kaplan-Meier method, and survival differences were assessed using the log-rank test. Variables suggested to be prognostic factors in univariate analysis (P<0.05) were subjected to multivariate analysis using a Cox proportional-hazards model. A P-value <0.05 was considered to indicate statistical significance. All statistical analyses were conducted using the SAS software package (JMP Pro11, SAS Institute, Cary, NC).

## Results

### Background of the primary GBC patients

The average age was 67 years (range, 40–86). Seventy-five (76%) of the 99 GBC patients underwent curative surgical resection (R0). Recurrence rate of the 99 primary GBC patients was 48%, and mortality was 46%. The median of postoperative follow-up period was 32 months (range, 0–243 months).

### *CDO1* promoter methylation level and its correlation with clinicopathological factors in GBC

**Fig 1:**
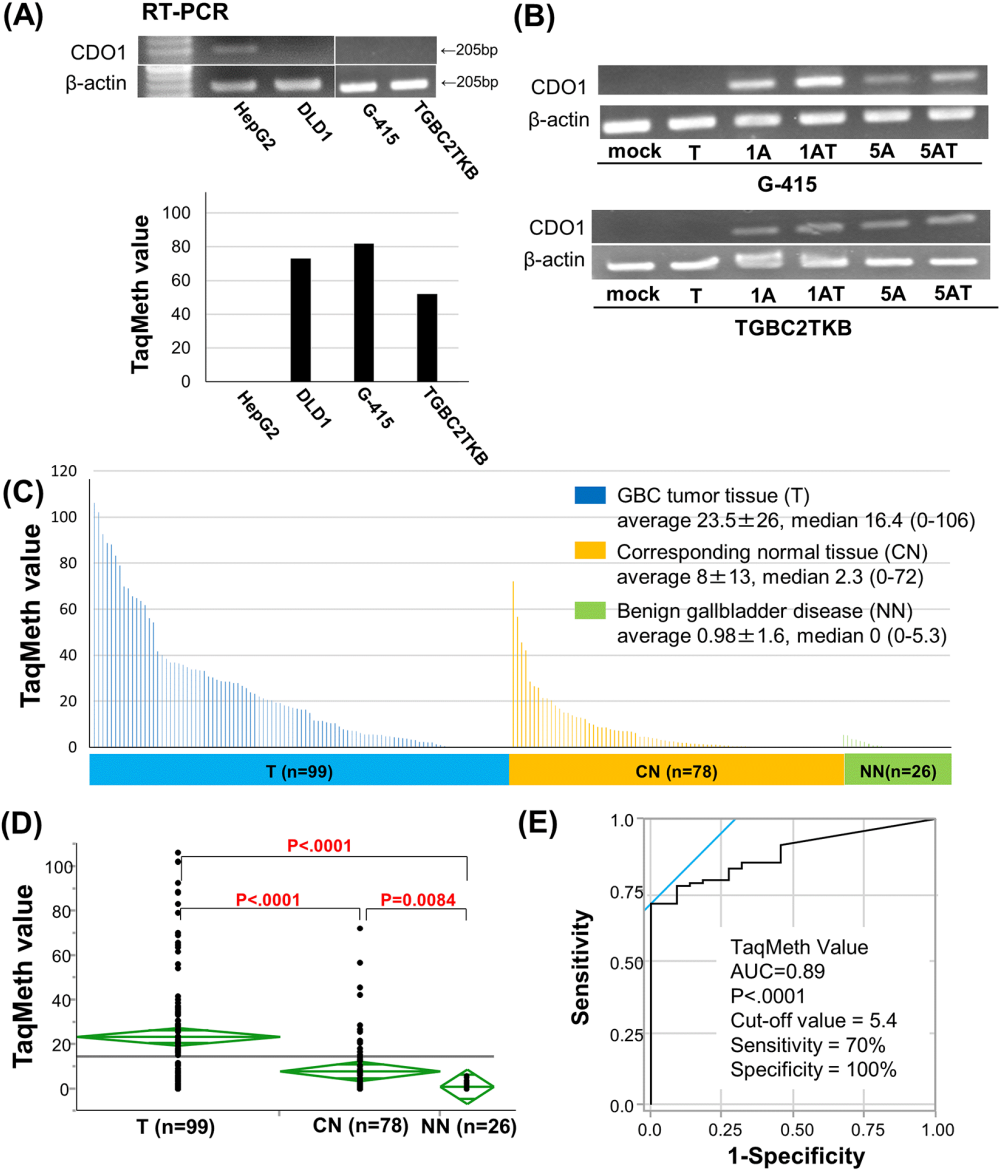
Quantitative assessment of *CDO1* methylation in primary GBC. (A) Expression level of CDO1 by RT–PCR (top panel) and CDO1 TaqMeth value in the GBC cell lines (G-415 and TGBC2TKB) (bottom panel). HepG2 cells, positive control for CDO1 expression; DLD1 cells, negative control for CDO1 expression. (B) Expression level after treatment with 5-Aza-dC alone, TSA alone or the combination by RT–PCR in the GBC cell lines. M, mock including the same volume of acetic acid; A, 5-Aza-dC (1 or 5 mM; 1A or 5A); T, TSA. (C) TaqMeth value in the 99 primary GBC tumor tissues (T), 78 corresponding normal tissues (CN) and 26 benign gallbladder disease (NN). (D) There was a significant difference in CDO1 TaqMeth values between T and CN (p = 0.0017), and between T and NN (p < .0001). (E) Receiver-operating characteristic curve of CDO1 methylation for the detection of primary gallbladder cancer. The area under the curve (AUC) represents the accuracy in discriminating T from NN in terms of its sensitivity and specificity (P < .0001).

In the 2 GBC cell lines, no basal expression of *CDO1* was seen (Fig 1A), and silenced expression of *CDO1* gene was robustly reactivated after treatment with the demethylating agent, 5-Aza-dC, 5-Aza-dC and TSA (Fig 1B). Furthermore, *CDO1* TaqMeth value in the GBC cell lines was detected highly as well as those of positive control DLD1 (Fig 1A). These results indicated that *CDO1* is epigenetically inactivated in GBC cell lines.

The median TaqMeth value in the 99 primary GBC tumor tissues (T) was 16.5, with values ranging from 0 to 106 and the average methylation of them was 23.5 ± 26, while the median methylation value in corresponding normal tissues (CN) of 78 was 2.3, with values ranging from 0 to 72 and the average methylation of them was 8 ± 13. The median value in diseased gallbladder tissues (NN) of benign gallbladder disease patients was 0, with values ranging from 0 to 5.3 and the average methylation of them was 0.98 ± 1.6 (Fig 1C). There was a significant difference in *CDO1* TaqMeth value between T and CN (p < .0001), and between T and NN (p < .0001) (Fig 1D). Promoter DNA methylation of the CDO1 gene showed highly discriminative receiver–operator characteristic (ROC) curve profiles, distinguishing T from NN (Fig 1E); the area under the ROC curve (AUROC) that discriminates T from NN was 0.89. In order to maximize sensitivity and specificity of detection, the optimal cut-off for methylation of the CDO1 gene was calculated from the ROC analysis (value 5.4). Using this cut-off, the sensitivity of the assay for GBC was 70%, while the specificity was 100%. In NN, *CDO1* TaqMeth value of XGc (median 2.7, range 0–5.3) was significantly higher than those of others (p = 0.0010) (S2A Fig).

**Fig 2:**
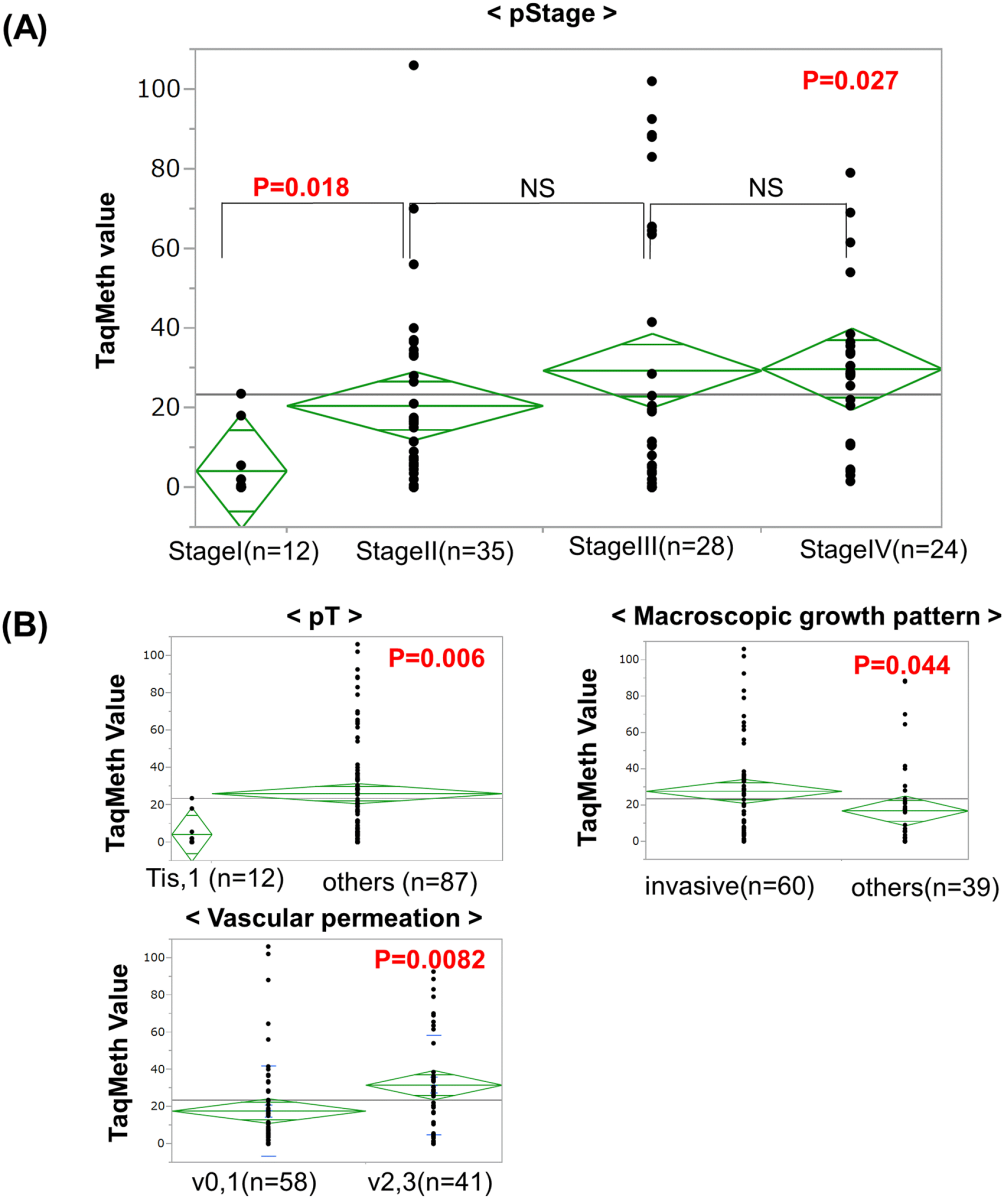
Correlation of the TaqMeth value of *CDO1* to various clinicopathological factors in primary GBC. **(A)** Correlation of the *CDO1* TaqMeth value to pStage. The TaqMeth values of the CDO1 gene showed significant differences between Stage I and other stages of GBC (p = 0.018). (**B)** Correlation of the *CDO1* TaqMeth value to clinicopathological factors other than pStage. The *CDO1* TaqMeth value was significantly associated with pT, macroscopic growth pattern and vascular permeation.

Correlation of each clinicopathological factor with *CDO1* TaqMeth values of the primary GBC was determined by using Student’s *t*-test, and is shown in Fig 2. There were significant correlations of *CDO1* TaqMeth values with pStage (p = 0.027), primary tumor factor (pT) (p = 0.0060), macroscopic growth pattern (p = 0.044), and vascular permeation (p = 0.0082). The other clinicopathological factors, such as preoperative serum values of CA19-9, lymph node metastasis, distant metastasis, and lymphatic permeation, were not significantly related to *CDO1* TaqMeth values based on analysis of variance (ANOVA).

**Fig 3:**
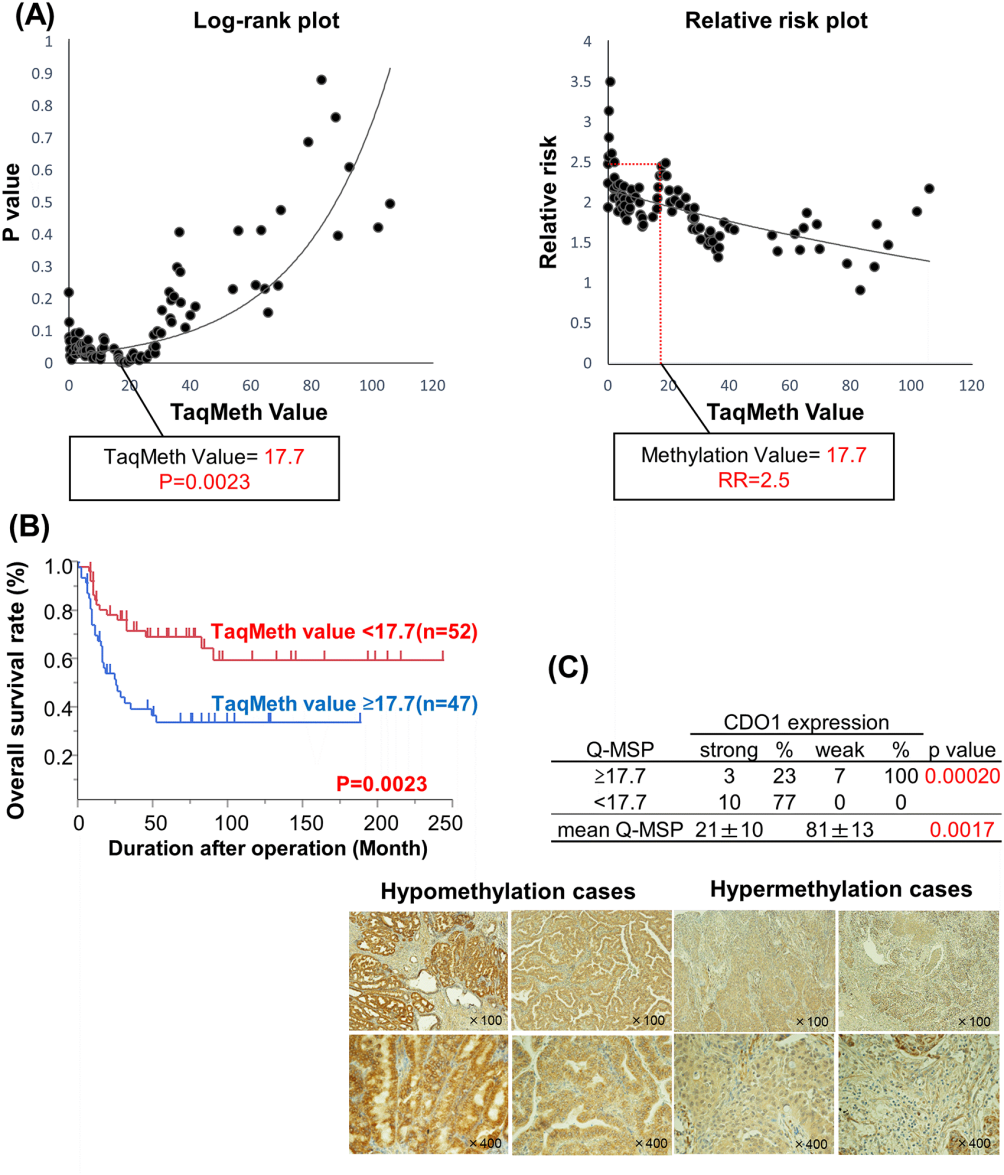
Prognostic analysis and CDO1 protein expression according to the TaqMeth value of *CDO1* in primary GBC. **(A)** Identification of an optimal cut-off value for the prognosis using the log-rank prognostic analysis. (B) Kaplan–Meier curves for CDO1 methylation status with value above or below 17.7 in the primary GBC (p = 0.0023). (C) Correlation between CDO1 protein expression and TaqMeth value in 20 primary GBC patients (top). Representative pictures taken from immunohistochemistry of CDO1 in methylation-positive or negative GBC samples (bottom left and right).

We further investigated whether the *CDO1* TaqMeth value could predict prognostic outcomes of GBC. A Kaplan-Meier curve of overall survival was constructed for the 99 patients according to the *CDO1* TaqMeth values, and p value and relative risk were each plotted to analyze survival differences between *CDO1* TaqMeth values above and below the best optimized cut-off determined by using the log rank test (Fig 3A). We have herein based on the methods to determine the best optimal cut-off value for the prognostic stratification using the log-rank prognostic analysis [15–17]. We thereby defined the optimal cut-off value of the *CDO1* TaqMeth value as 17.7 which showed the highest relative risk with statistical significance (p<0.05). Using this cut-off value to divide the patients into hyper- and hypo-methylation groups, the hypermethylation group showed a survival rate of 34% (n = 47), while the hypomethylation group showed a survival rate of 69% (n = 52). The prognostic difference between these two groups showed high statistical significance (p = 0.0023) (Fig 3B).

We then examined the differences of CDO1 protein expression in GBC tissues by immunohistochemistry with anti CDO1 polyclonal antibody, where patients with CDO1 hypomethylation (n = 10) and CDO1 hypermethylation (n = 10) were compared. Intense expression of CDO1 protein was observed in all CDO1 hypomethylation cased (n = 10), whereas weak expression of CDO1 protein was dominant in seven cases (70%) of CDO1 hypermethylation cases (n = 10). The difference of CDO1 protein expression between these two groups showed statistical significance (p = 0.0002) (Fig 3C).

### Univariate and multivariate prognostic analyses including CDO1 TaqMeth values in primary GBC

The 5-years OS rate of all GBC patients was 52%. The 5-years OS in each stage was 92%, 78%, 36%, and 13%, in Stage 0/I, II, III, and IV, respectively (S1 Fig). Univariate prognostic analyses showed that preoperative serum value of CA19-9, preoperative inflammation, primary tumor factor (pT factor), lymph node metastasis (pN factor), distant metastasis (pM factor), stage, histology, mode of histological infiltration, macroscopic growth pattern, lymphatic permeation, vascular permeation, and resection status were factors indicative of poor prognosis (Table 1).

**Table 1:** Univariate and multivariate analysis for overall survival (OS).

Clinicopathological parameters	Number	Univariate analysis		Multiivariate analysis		
		OS (%)	p value^a^	HR	95%CI	p value^b^
Age						
<65 / ≥65	38 / 61	48 / 54	0.448			
Gender						
male / female	38 / 61	44 / 57	0.084			
Preopertive serum CA19-9						
<37 / ≥37	56 / 43	68 / 32	0.0001	1.7	0.84–3.7	0.05
Preoperative inflammation						
absence / presence	83 / 16	58 / 25	0.013	1.6	0.53–5.8	0.40
Preoperative jaundice						
absence / presence	91 / 8.	54 / 38	0.46			
Preoperative biliary drainage						
absence / presence	86 / 13	58 / 15	0.0007	2.1	0.56–8.0	0.27
Tumor location						
Gfb / GnC	64 / 35	53 / 49	0.68			
Histology						
well, mod, pap / others	74 / 25	62 / 24	0.0004	1.2	0.60–2.4	0.58
Mode of histological Infiltration						
α / βγ	18 / 81	81 / 47	0.017	1.6	0.42–8.1	0.52
Macroscopic growth pattern						
others / invasive	39 / 60	68 / 42	0.0082	0.63	0.27–1.5	0.29
Lymphatic permeation (ly)						
ly0, 1 / ly2, 3	73 / 26	69. / 4	<.0001	2.4	1.1–5.5	0.028
Vascular permeation (v)						
v0, 1 / v2, 3	58 / 41	71 / 25	<.0001	1.33	0.54–3.2	0.53
Operative procedures						
SCx / SR / ER	36 / 47 / 16	43 / 63 / 41	0.092			
Lymph node dissection (D)						
D1, 2 / D0	74 / 25	57 / 38	0.060			
Resection status (R)						
R0 / R1, 2	75 / 24	67. / 6	<.0001	2.6	1.0–6.5	0.042
Postoperative chemotherapy						
absence / presence	51 / 48	59 / 45	0.23			
TaqMeth Value						
<17.7 / ≥17.7	52 / 47	69 / 34	0.0023	2.1	1.0–4.4	0.047
pStage*						
I	12	92	<.0001	Reference		0.54
II	35	78		2.2	0.30–45	
III	28	36		3.7	0.53–77	
IV	24	13		3.8	0.44–86	

Abbreviations: HR, Hazard Ratio; CI, confidence interval; Gfb, fundus and body of gallbladder; GnC, gallbladder neck and cystic duct; SCx, simple cholecystectomy; SR, standard resection; ER, extended resection.

* Classification of biliary tract cancers established by Japanease Society of Hepato-Biliary Pancreatic Surgery (JSHBPS): The 6^th^ edition.

^a^ Log-rank test.

^b^ Cox-proportional hazard model.

The clinicopathological factors related to prognosis were then applied to a multivariate Cox proportional hazards model. Individual TNM factors were excluded, because they are considered to be confounding factors. The multivariate analyses indicated that *CDO1* DNA hypermethylation (p = 0.047), lymphatic permeation (ly2/3) (p = 0.028), and resection status (R1/2) (p = 0.042) were independent prognostic factors that were significantly related to OS in primary GBC (Table 1), and stage was eliminated as a prognostic factor. Kaplan-Meier curves of the overall survival of the patients according to the independent prognostic factors of lymphatic permeation and resection status are shown (S1B Fig).

### Correlation of clinicopathological factors to the CDO1 TaqMeth value status divided by the prognostically optimized cut-off value in primary GBC

Correlation of clinicopathological factors in primary GBC according to *CDO1* promoter DNA methylation status, using a *CDO1* TaqMeth cut-off value 17.7, was determined using a χ^2^ test. pT factor (≥T2 advanced), advanced stage, mode of histological infiltration (β/γ), lymphatic permeation (ly2/3), and vascular permeation (v2/3) were significantly related to *CDO1* promotor DNA hypermethylation status (Table 2).

**Table 2:** Correlation of clinicopathological factors and *CDO1* methylation.

Clinicopathological parameters		CDO1 TaqMeth value				p value^a^
		low (<17.7)		high (≥17.7)		
		n = 52		n = 47		
		No.	%	No.	%	
**Preoperative factor**						
Age	<65	22	42	16	34	0.40
	≥65	30	58	31	66	
Gender	male	22	42	16	34	0.40
	female	30	58	31	66	
Preopertive serum CA19-9	<37	30	58	26	55	0.81
	≥37	22	42	21	45	
Preoperative inflammation	absence	44	85	39	83	0.83
	presence	8	15	8	17	
Preoperative jaundice	absence	47	90	44	94	0.56
	presence	5	10	3	6	
Preoperative biliary drainage	absence	44	85	42	89	0.49
	presence	8	15	5	11	
**Pathological factor**						
pT	Tis, T1	10	19	2	4	0.023
	other	42	81	45	96	
pN	N0	37	71	28	60	0.23
	N1	15	29	19	40	
pM	M0	46	88	37	79	0.19
	M1	6	12	10	21	
pStage*	I	10	19	2	4	0.023
	others	42	81	45	96	
Tumor location	Gfb	35	67	29	62	0.56
	GnC	17	33	18	38	
Histology	well, mod, pap	42	81	32	68	0.15
	other	10	19	15	32	
Mode of histological Infiltration	α	14	27	4	9	0.018
	β, γ	38	73	43	91	
Macroscopic growth pattern	invasive	27	52	33	70	0.063
	others	25	48	14	30	
Lymphatic permeation (ly)	ly0, 1	44	85	29	62	0.0097
	ly2, 3	8	15	18	38	
Vascular permeation (v)	v0, 1	38	73	20	43	0.0021
	v2, 3	14	27	27	57	
**Treatment factor**						
Operative procedures	Standard resection	26	50	20	43	0.46
	others	26	50	27	57	
Lymph node dissection (D)	D0	15	29	16	34	0.99
	D1, 2	27	52	29	62	
Resection status (R)	R0	42	81	33	70	0.22
	R1, 2	10	19	14	30	
Postoperative chemotherapy	absence	26	50	25	53	0.75
	presence	26	50	22	47	

Abbreviations: Gfb, fundus and body of gallbladder; GnC, gallbladder neck and cystic duct

* Classification of biliary tract cancers established by Japanease Society of Hepato-Biliary Pancreatic Surgery (JSHBPS): The 6^th^ edition.

^a^ χ2 test or Fisher exact test

### Prognostic relevance of the CDO1 TaqMeth value in each stage of GBC

**Fig 4:**
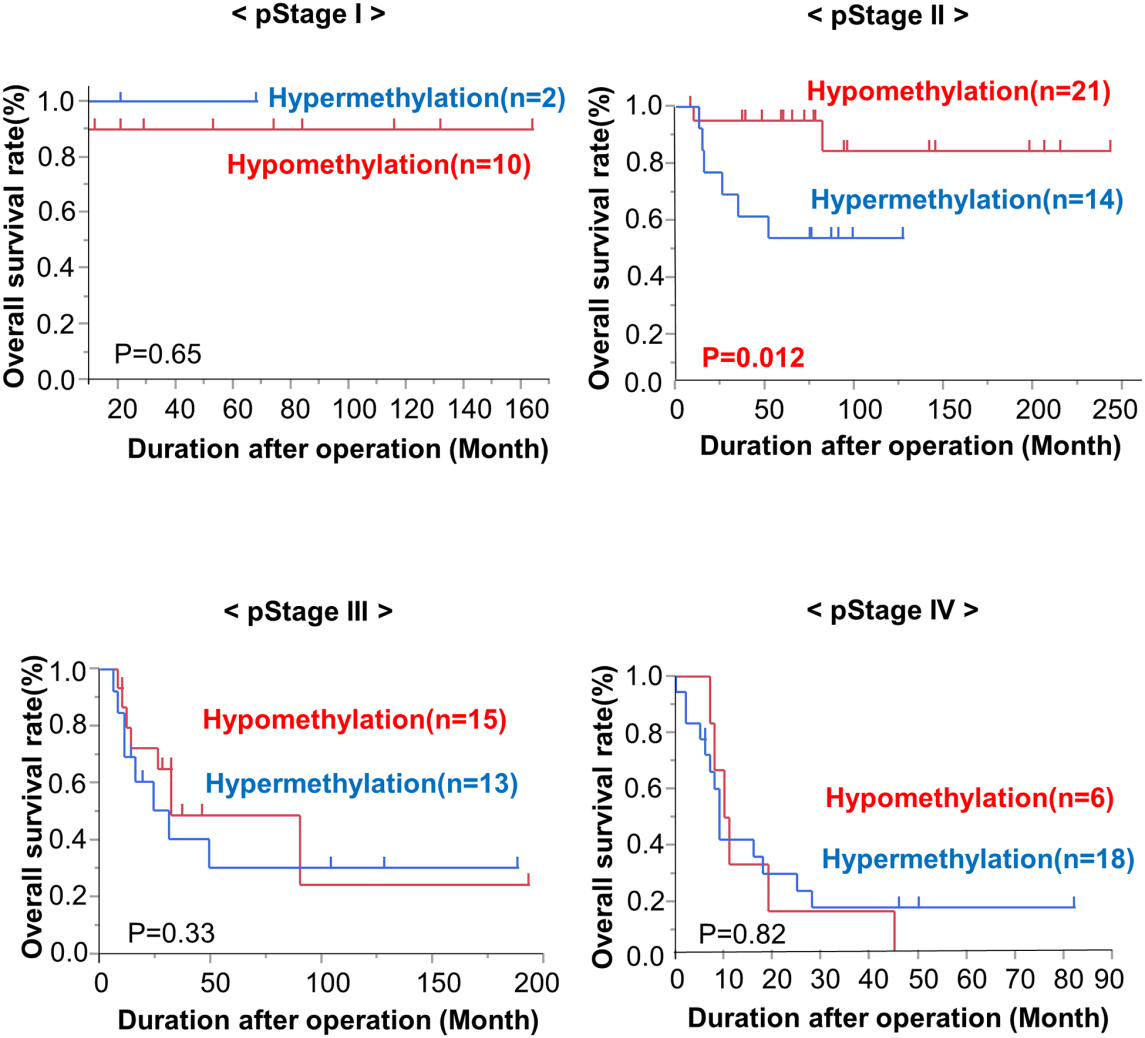
Kaplan-Meier curve for overall survival of each pathological stage according to the *CDO1* TaqMeth value. The CDO1 gene hypermethylation group showed poorer prognosis than the CDO1 gene hypomethylation group, especially in Stage II (p = 0.023), but not in other Stages.

The TaqMeth values of the *CDO1* gene showed significant differences between Stage I and other stages of GBC (Fig 2B). We also examined the OS of each pStage (stage I, stage II, stage III and stage IV) according to *CDO1* TaqMeth values. This analysis showed that the hypermethylation group showed poorer prognosis than the hypomethylation group, especially in Stage II (p = 0.023) (Fig 4). In addition, we performed a subgroup prognostic analysis for stage II GBC CDO1 hypomethylation group vs. CDO1 hypermethylation group. Lymphatic permeation (ly2/3) was significantly related to CDO1 hypermethylation status in Stage II (p = 0015).

## Discussion

We recently confirmed the CDO1 gene as being specifically methylated in various human cancers [8] following our development of an original algorithm designated as a pharmacological unmasking microarray (PUM), which suggested that *CDO1* plays a tumor suppressive role in human carcinogenesis [5]. Promoter DNA of the CDO1 gene has been reported to be frequently methylated in various cancers, including breast [6–9], esophagus [11], lung [8, 18, 19], bladder [8], gastric [8], colorectal [8, 20], biliary tract [13], hepatocyte [21], renal clear-cell cancer [10], and testicular germ cell cancers [22]. Furthermore, we previously described detection of *CDO1* methylation in the plasma of colorectal cancer (CRC) using methylation specific PCR (Q-MSP) and extensive analysis of the PCR reaction [23]. We showed that analysis of plasma *CDO1* methylation in combination with CEA/CA19-9 levels increases the detection rate of curable CRC patients. In the present study, we investigated for the first time the promoter DNA methylation status of the CDO1 gene in primary GBC. We found that *CDO1* promoter hypermethylation was cancer prone and strongly associated with tumor malignancy. However, *CDO1* TaqMeth value of CN was significantly higher than those of NN (p = 0.0084), and the AUC of *CDO1* methylation to discriminate T from CN was 0.71, which is the lowest value compared to those reported to date in the literature for cancers such as breast (AUC, 0.84), esophagus (AUC, 0.91), lung (AUC, 0.87), bladder (AUC, 0.87), and stomach (AUC, 0.95) [8]. Furthermore, TaqMeth value of CN with *CDO1* hypermethylation status in T was significantly higher than them of CN with *CDO1* hypomethylation status in T by the χ^2^ test (p = 0.011). Frequent *CDO1* methylation of the CN may suggest the existence of a potentially precancerous lesion around the main lesion.

Interestingly, *CDO1* hypermethylation was also found in xanthogranulomatous cholecystitis (S2A Fig). Xanthogranulomatous cholecystitis (XGC) is an uncommon variant of chronic cholecystitis characterized by xanthogranulomatous inflammation of the gallbladder. Intramural accumulation of lipid-laden macrophages and acute and chronic inflammatory cells is the hallmark of the disease. The xanthogranulomatous inflammation of the gallbladder can be very severe and can spill over to the neighbouring structures like liver, bowel and stomach resulting in dense adhesions, perforation, abscess formation, fistulous communication with adjacent bowel. Striking gallbladder wall thickening and dense local adhesions can be easily mistaken for carcinoma of the gallbladder, both intraoperatively as well as on preoperative imaging [24]. In addition, although XGC is not believed to be a premalignant lesion, the frequency of coexisting XGC and GB cancer is nearly 10% [25]. There were no patients with coexisting XGC and GB cancer in this study, but this result propose further study whether aberrant *CDO1* DNA methylation in XGC mean possibility to be an onset factor of GBC or the result of accumulation of severe inflammation of the gallbladder wall.

There was no significant difference in *CDO1* methylation between Stage0/I and the corresponding non-cancerous mucosa tissues (CN) (S2B Fig). However, the *CDO1* methylation value increased in a stepwise manner towards advanced stages of GBC; in particular there was a significant difference in *CDO1* methylation between Stage I and Stage II (p = 0.018) (Fig 2A). Prognosis of Stage I was very much better than that of Stage II (S1A Fig) as reported previously [26, 27]. The gallbladder has unique histological characteristics including the fact that it does not have lamina muscularis mucosae and that cancer cells tend to more easily infiltrate into the thin intrinsic muscle layer and serosa in comparison with other cancers. That is the reason why the increase in *CDO1* methylation from Stage I to Stage II is important.

More importantly, we also showed that *CDO1* hypermethylation was significantly associated with poor prognosis in Stage II GBC patients, where all patients underwent R0 resection, and when the optimal cut-off value of *CDO1* methylation was determined according to the log-rank plot analysis for total cases. This finding indicated that the *CDO1* methylation cut-off value of 17.7 has clinically meaningful significance, because the stage II patients with a *CDO1* methylation value below 17.7 showed excellent prognosis that was similar to that of stage I patients. There have been numerous clinical reports describing that GBC patients with either Stage I or Stage II are potentially resectable with radical surgery and have a reasonable prognosis, but that some of the Stage II patients showed a poor prognosis. When postoperative surveillance strategy including adaptation of postoperative adjuvant therapy is considered, *CDO1* hypermethylation could be a beneficial predictor for patients at risk for recurrence. Moreover, in the log-rank plot analysis of the *CDO1* TaqMeth value, the higher this value was, the worse the prognosis was, and this held true for almost all cut-off values among the TaqMeth value range from 1 to 30. Therefore, *CDO1* methylation status is regarded as an ideal prognostic indicator of primary GBC.

In this study, *CDO1* hypermethylation was significantly associated with GBC tumor progression parameters, if the *CDO1* methylation cut-off value of 17.7, which was meaningful from a prognostic point of view, was used. These findings suggested that *CDO1* methylation was significantly associated with phenotypic acquisition of invasive and metastatic properties in primary GBC. Brait et al. reported that forced expression of full-length *CDO1* in human cancer cells markedly decreased anchorage independent growth and/or tumorigenesis even in a mouse model, whereas knockdown of *CDO1* inversely increased cell growth in both states. These functional data suggested that *CDO1* hypermethylation may play a causative role in tumor progression of primary GBC, rather than in the results of progression.

## Limitation

The limitations of this study included a retrospective study over 20 years with 99 GBC patients and loss of statistical power in the stage-specific prognostic sub analysis. So, this study might suffer from bias, i.e., change in treatment strategy such as range of lymph node dissection and application of chemoradiotherapy. Prospective validation is further needed to clarify the relationship between CDO1 promotor DNA methylation and prognosis in primary gallbladder cancer.

## Conclusions

Promoter DNA methylation of the *CDO1* gene was, for the first time, proven to be cancer-prone in primary GBC, and it could be a useful biomarker independent of stage in primary GBC.

## Supporting information

S1 Fig Kaplan-Meier curve for overall survival of pStage and independent prognostic factors. **(A)** Comparison of overall survival according to pStage. **(B)** The Kaplan-Meier curve of the overall survival of the patients are shown according to lymphatic permeation and resection status.
(TIF)

S2 Fig Comparison of the TaqMeth value of the CDO1 gene in various gallbladder disease. **(A)** In the diseased gallbladder tissues (NN) of benign gallbladder disease patients, *CDO1* TaqMeth value of XGc was significantly higher than those of others (p = 0.0010). **(B)** There was no significant difference in *CDO1* methylation between tumor tissues (T) of Stage 0, I patients and the corresponding non-cancerous mucosa tissues.
(TIF)

S1 Table PCR production and sequence of primers and fluorescent probe. (DOCX)
