# Supplementary material for: Prognostic significance of promoter DNA hypermethylation of the cysteine dioxygenase 1 (CDO1) gene in primary gallbladder cancer and gallbladder disease
Source: PLoS One. 2017 Nov 21;12(11):e0188178. doi: 10.1371/journal.pone.0188178 (PMC5697808; doi:10.1371/journal.pone.0188178)
Supplement: Supplementary file 1 [file pone.0188178.s003.docx]

Table S1. PCR production and sequence of primers and fluorescent probe

Gene	Method		Sequence
**CDO1**	**Q-MSP** *1	**Forward primer (5'>3')**	CCACAACGACGAAAATAAAACG
		**Fluorescent probe (5'>3')**	FAM-TTAACGGCGCGTTTTAGTCGTTCG-TAMRA
		**Reverse promer (5'>3')**	TCGGCGTTTTAGGGATCGCG
**βactin**	**Q-MSP** *1	**Forward primer (5'>3')**	TGGTGATGGAGGAGGTTTAGTAAGT
		**Fluorescent probe (5'>3')**	FAM-ACCACCACCCAACACACAATAACAAACACA-TA
		**Reverse promer (5'>3')**	AACCAATAAAACCTACTCCTCCCTTAA
**CDO1**	**RT-PCR**	**Forward primer (5'>3')**	GAGGGAAAACCAGTGTGCCT
		**Reverse promer (5'>3')**	GCCCGAAGTTGCATTTGGAG
**βactin**	**RT-PCR**	**Forward primer (5'>3')**	TCACCCACACTGTGCCCATCTACGA
		**Reverse promer (5'>3')**	CAGCGGAACCGCTCATTGCCAATGG

*1 : Q-MSP was done at 95° C for 3 min followed by 40 cycles at 95° C for 20 sec, 60° C for 30 sec, and 72°
in a 25μl reaction volume containing 100ng treaated DNA, 300nmol/l each primer, 200nmol/l fluorescect probe
iQTM supermix.
The forward and reverse of primers for Q-MSP were designed within 312 bp and 223 bp proximal from the TSS
CDO1 promoter the region which covered most of the CpG rich region.
The probe for Q-MSP was designed within 274 bp proximal from the TSS in the CDO1 promoter the region.
